# Multilevel Evaluation of ECHO Autism Programs: Development and Pilot Study of a Standardized Minimum Dataset

**DOI:** 10.2196/86274

**Published:** 2026-08-18

**Authors:** Katrina Boles, Kimberly Hoffman, Melissa Mahurin, Robin Hardesty, Valeria Nanclares-Nogués, Melinda Odum, Ramiro Mitre, Kristin Sohl

**Affiliations:** 1 Department of Pediatrics, ECHO Autism Communities, School of Medicine, University of Missouri, 400 N Keene St, Columbia, MO, 65201, United States, 1 5735290035; 2 Family and Community Medicine, School of Medicine, University of Missouri, Columbia, MO, United States; 3 Fundación Neurodiversidad, Rosario, Argentina

**Keywords:** program evaluation, learning health system, training support, autism spectrum disorder, virtual learning models, Project ECHO

## Abstract

**Background:**

The rising prevalence of autism highlights the challenges faced by health care professionals, educators, and guardians in managing autism care. ECHO (Extension for Community Healthcare Outcomes) Autism Communities was established to train professionals in best practice autism care and diagnosis through regular virtual case-based learning sessions. As programmatic offerings expanded to include more professions and ongoing care after diagnosis, program measures were adapted to fit the desired learning outcomes and were adapted over time. As a result, consistent measures were not in place to assess any individual program longitudinally, and assessments of all programs collectively were even further limited. The global expansion of the ECHO Autism model led to an insistent need for standardized outcome measures to evaluate effectiveness.

**Objective:**

In this study, a team consisting of ECHO Autism leaders with more than 20 years of combined knowledge of ECHO Autism programs reviewed existing measures to create a standard set of participant survey metrics, the ECHO Autism Communities minimum dataset (MDS). The study included a rapid 3-month pilot with a small 3-program dataset, followed by a 2-year pilot across 8 programs.

**Methods:**

The study team collected 42 surveys from existing programs, organized all survey metrics, and refined them into a standardized set of metrics. The MDS was designed to measure priority information, set consistent scales and anchors, use inclusive language, standardize response choices, and determine units of analysis to transform data into actionable information. An early version of the MDS was translated into Spanish and implemented as a 3-month pilot across programs in the United States, Argentina, and Chile. Participant program-level data for the 2-year pilot were visualized and presented in PDF reports. Wilcoxon signed-rank tests of significance were performed on postsurvey self-efficacy data for programs and across all programs. An interactive dashboard was created for comparisons.

**Results:**

Three-month pilot data provided actionable insight so that adjustments were made before ongoing evaluation. The first 2 years of MDS implementation showed consistency in evaluation and significant improvements in self-efficacy across 8 programs. ECHO Autism leaders appreciated clear and simple data visualizations that showcased aggregate participant demographics, as well as changes in self-efficacy and barriers.

**Conclusions:**

As a result of this study, ECHO Autism leaders can assess and compare program effectiveness, participant characteristics, and demonstrate the program’s impact on professionals providing autism care. This study highlights the importance of consistent outcome measures for evaluation and enabling data-informed decisions for similar professional training programs for delivering autism best practices.

## Introduction

### Background

As the fastest-growing developmental condition in the United States, autism has seen a prevalence increase from 1 in 150 to 1 in 31 in 20 years [1-3]. Health care professionals face several challenges in their experiences with autism, including lack of training and knowledge to address the complexities of autism and co-occurring conditions [4]. Guardians of young children suspected of having autism face several challenges in receiving diagnosis and ongoing management of care, including delays in diagnosis [5], provider shortages [6], lack of mandated autism training in health care programs [7], and navigating complicated eligibility criteria for services [8].

Continuing education (CE) programs are intended to provide additional training to professionals in areas of need, such as autism. One such model for CE training is Project ECHO (Extension for Community Healthcare Outcomes), which was established in 2003 to expand knowledge to community health care professionals [9-11]. The ECHO model connects multidisciplinary expert specialists (a hub team) with geographically dispersed professionals (participants) to distribute best-practice knowledge in specialized care through regular virtual meetings (ECHO sessions). ECHO sessions are part of a year-long or shorter series often recurring at 1- or 2-week intervals, a defined timeframe for an individual program. Programs have a defined target audience, learning objectives, and a multidisciplinary hub team with expertise to mentor participants within the learning objectives. An example hub team makeup for a program focused on autism would include autism experts from some of the following fields: pediatrics, psychology, psychiatry, social work, speech-language pathology, nutrition, education, or occupational therapy. In addition, each hub team would include at least one expert by lived experience, for example, the parent of an autistic child [12,13]. Each ECHO session includes a short learning didactic presented by a hub expert and a deidentified case presented by a participant, providing real-world examples for group learning and discussion among all learners. While considerable research and publications have been produced from ECHO programs [14], the program evaluation metrics are inconsistent. Additionally, previous research has indicated a need for more rigor in quantifying the value of ECHO programs, especially in determining the effects on changes in practice [15,16].

There are several advantages to using consistent outcome measures for evaluating ECHO programs. When multiple programs use the same measures, it becomes possible to assess the effectiveness of the entire organization’s programs. Consistent longitudinal measurement makes it possible to evaluate trends, determine the fidelity of programs in accomplishing their missions, and modify programs to continuously improve. And finally, data analysis and reporting can be streamlined for faster dissemination and action. Hub team members gain insight to adjust teaching, and the organization’s leadership team keeps their fingers on the pulse of all the programs.

There are many examples of organizations that have developed and use consistent measures in health care; they include accreditation agencies, research collaboratives, and registries. Some of the better-known standardized metrics are the quality indicators from the Agency for Healthcare Research and Quality (AHRQ), which are designed to identify quality-of-care concerns in hospital performance administrative records [17]. The clinical trials registry at clinicaltrials.gov includes mandatory data collection that makes it possible to perform searches to connect individuals to trials and vice versa [18]. The Cystic Fibrosis Foundation patient registry was developed to collect consistent data for use in research, clinical care, and tracking the temporal landscape of the disease [19]. Cincinnati Children’s Hospital uses evidence-based care guidelines they developed to focus organizational change around key performance data [20,21].

There are many challenges in standardizing learning and behavioral measures across programs that have differing audiences, foci, and learning outcomes. One challenge is the changing landscape of best practices learned within programs. Another challenge is that curriculum is program- and audience-specific. Reliable measures for evaluating behavior change may not exist depending on the domain due to challenges in accessing patient charts and a lack of standardized records across disciplines. Despite the challenges in gathering consistent unbiased behavioral data, there are other methods for assessing behavioral change that could be applied within a standardized metric, such as participant surveys. Though it is not without limitations, analysis of self-identified behavioral changes provides an informative assessment of changes in practice. At the start of this study, no standardized outcomes and measurements for longitudinal training in autism and developmental disabilities were available to ECHO programs.

### Prior Work

In 2015, medical researchers at the University of Missouri founded ECHO Autism Communities (EAC) to apply the ECHO model to autism [9,10]. Prior to this study, EAC had collected several years’ worth of participant surveys for multiple centrally run ECHO programs. Programs were offered for various audiences and based on topics, such as diagnosis, behavior management, and advocacy. These data were usually collected before and after participation. Evaluation of program success included participant-reported satisfaction, questions about barriers individuals face in their work with autism, and self-efficacy. Due to changes in survey questions following each program cycle, evaluation was limited to assessment by participant cohorts for each program.

In 2021, EAC became an authorized trainer to support organizations launching autism and developmental disabilities ECHO programs worldwide. This outreach has resulted in more than 92 program launches in 24 countries by partner organizations. A need for harmonized measures became critical to provide a higher level of reporting so that data-informed decisions could be made across all programs and partner organizations.

### Study Objectives

In this study, we created a standard set of participant survey metrics that would (1) allow measurement of priority information across all partner sites and programs, (2) set consistent scales and anchors, (3) use inclusive language, (4) standardize response choices where possible, and (5) predetermine unit(s) of analysis to transform data into actionable information. Data from a pilot study and the first 2 years’ results from the ECHO Autism Communities minimum dataset (MDS) were used to assess the usability and value of the MDS.

## Methods

### Guiding Principles

We used several guiding principles in our work to create the MDS. First, we wanted to create a line of sight between the evaluation of programs and the mission and values of the EAC organization. The EAC mission states: “ECHO Autism is committed to building global communities where people see possibilities in all abilities.” As an organization, our values include (1) access to equitable best practices; (2) person-family-centered care; (3) embrace curiosity; (4) all teach, all learn; and (5) see the possibilities in all abilities. One example of this guiding principle in action included constant review of the language in our questions from a global perspective to assess if they were respectful to communities outside the United States [22]. Second, we recognized that these surveys would be used across all programs and could not reasonably cover everything an individual program would need. Third, we wished to maintain consistency, including the language and the scales used in choice answers. Fourth, it was important that we create a process that could be sustained over time. We took 2 actions to streamline analysis: (1) we reviewed previous analyses of open-ended responses to build choice answers to questions rather than allowing free-text responses and (2) we carefully considered which questions should have an optional “other” choice where respondents could write in answers. Our fifth guiding principle was to transform data into actionable information to continuously enhance programs.

### Ethical Considerations

This project was conducted as a within-team quality improvement evaluation using existing program metrics and previously collected data. Data included in this analysis were collected under multiple quality improvement projects, reviewed and approved by the Institutional Review Board (IRB) at the University of Missouri, in accordance with ethical standards for quality improvement initiatives. No new identifiable data were collected for the purposes of this study, and all analyses were conducted on deidentified or aggregated data under IRB #2015762-QI.

### Study Design

**Figure 1.**
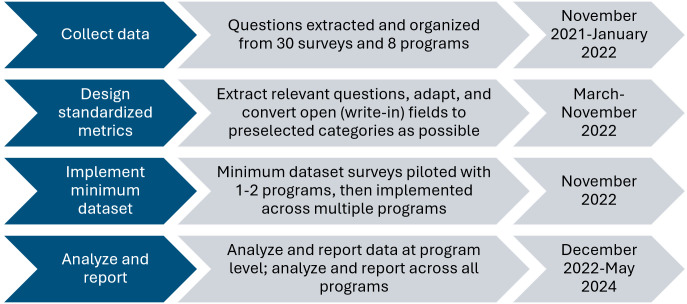
Process and timeline for developing a minimum dataset.

Work began to develop the MDS in 2021. The study team included directors for EAC (n=3) overseeing replication, data, and global expansion; an emerita faculty member; a data analyst; and the EAC executive director. Expertise of the study team spans decades and includes education, evaluation metrics development, psychology, medicine, informatics, user-centered design, and autism diagnosis and management. The process for developing and implementing the MDS included data collection, metric design, implementation, and reporting (Figure 1).

### Collect Existing Surveys

At the start of this study, the team gathered 30 existing presurveys and 12 existing postsurveys used by 8 programs. This yielded 761 total unique questions. Questions were extracted from the surveys and combined, organized based on similarity, and categorized (eg, demographics, practice patterns, self-efficacy). Radio button and checkbox choices were included in this process.

### Design Standardized Metrics

Over 6 meetings, the study team reviewed program surveys and other quality-improvement surveys to standardize wording of questions and choice answers. The review included the following categories: participant contact information, organization information, demographics, prior knowledge and experience with autism, professional practice patterns, barriers, and motivation for participating.

Within the 761 questions, we reviewed each category and collectively assessed questions for relevance across all programs and audiences during collective team meetings. The team standardized similar questions and excluded questions that were audience- or program-specific. In review of open-ended questions, the team relied upon previous qualitative analyses of these data to define standardized choice responses. Drafts of the MDS were further examined by team members outside of the meetings, and further adjustments were brought back to the full group for discussion and unanimous agreement. Where unanimous agreement was not achievable, the Executive Director for ECHO Autism Communities adjudicated disputes.

The self-efficacy group required additional consideration and evaluation. Unlike other categories, self-efficacy statements were often program-specific and not easily transferable across all programs. We began with 306 self-efficacy statements and ended with 36, often making adaptations for relevance across all audiences. Statements were organized into categories around the EAC mission and values. We also reviewed previously used Likert scales. We determined that we would use a 5-point Likert scale from no confidence (0) to highly confident (4) for all 36 statements because it was our most-commonly used scale, and we would require respondents to rate each statement. We decided to allow participants to identify if there were statements that did not apply and asked them to explain why rather than offer N/A in the scale so that the “n” for each metric would not change. The team valued a retroactive pre-assessment (retro-pre) of self-efficacy at the end of programs, where participants are asked about their abilities before attending and after. The retro-pre was implemented to maximize retention of data. Additionally, the retro-pre allows respondents to determine how much they felt their self-efficacy changed and is a remedy for response shift bias that occurs in a true pre-post evaluation [23]. For this study, our pre-post data are not paired; however, the method of collecting both a true-pre and retro-pre as identifiable data enables the analysis of participant response bias. Collectively, the team unanimously agreed upon which metrics to adapt and include in the minimum dataset. Once the metrics were developed, the MDS was translated into Spanish.

### Implement Minimum Dataset

To gather preliminary data collection and to rapidly assess the results of most of the MDS, we completed a short-term pilot including 38 participants from multiple countries. In the fall of 2022, the MDS presurvey was used in a centrally run program with 19 participants primarily located in the United States and in 2 programs run by partner organizations with 19 Spanish-speaking participants located primarily in Argentina and Chile. All participants were asked to complete the presurvey in either English or Spanish as part of their registration. Data were collected and securely managed using REDCap instruments hosted at the University of Missouri [24,25]. Data collection was open only to registrants and participants of the programs. Only ECHO Autism employees have access to the REDCap data. Following this pilot, the team made slight adjustments to the MDS and then implemented the MDS across all centrally run EAC programs from 2023 to 2024. Program participants for 8 EAC programs were emailed a link to voluntarily complete a presurvey before participation and a postsurvey following program conclusion. Survey completion incentives were determined by the programs, with 2 programs offering small denomination e-gift card incentives for postsurvey completion.

### Analyze and Report

Analysis of pilot programs in the United States, Argentina, and Chile, and 2023‐2024 MDS data occurred at a programmatic level for each individual series. To achieve our goals of sustainability and creating actionable information, we removed multiple entries from individuals, keeping the first entry or most complete entry, created data visuals, and exported summary data statistics for each series. The participant survey results of each series were made into a PDF report for actionable review by the hub team and EAC leadership before more than 2 ECHO sessions had occurred. The hub team used these reports to understand the participants’ background and self-efficacy around the learning outcomes, enabling hub teams to cater the learning to their participants. Reports were updated with only participant data and postsurvey results after all data were collected and again provided to the program hub team and leaders to inform decisions for any future series. Program-specific questions were included in these reports. The results for each question were presented individually, so the “n” varied across reports, and missing data were not interpreted. Following best practices for design and data displays, we chose data visualizations for optimum clarity and rapid understanding [26-29]. Single-choice questions were visualized as bar graphs or pie charts; multiple-choice questions were visualized as bar graphs. We analyzed self-efficacy data from the perspective of all participants and presented the data as bar charts showing an average response for each category and for all statements, annotated with average percent change score, calculated as ((After Rating – Before Rating)/Maximum Possible Value) × 100. Incomplete self-efficacy data were removed from analysis.

In addition to series-specific reports for hub teams, MDS results from 2023 to 2024 pre- and postsurveys were aggregated across all centrally run EAC programs and presented in an interactive interface so that the units of analysis could be viewed (1) as a single series of a program (series level), (2) the program longitudinally including multiple series’ (program level), and (3) for all EAC programs (organization level). Additionally, we completed statistical analysis using Wilcoxon signed-rank tests and Wilcoxon effect size (*r*) with CIs to determine significance with an alpha of .05 for retro-pre self-efficacy data at the program level and the organization level using R Studio. Within each self-efficacy category, an average of all ratings was calculated for each individual respondent so that the “n” would remain constant across categories in the tests. Only responses with complete self-efficacy values across all domains were included in statistical analysis. Prior to conducting Wilcoxon signed-rank tests, all zero differences were removed, and the total number of zero differences were reported.

## Results

### Standardized Metrics

The MDS has 122 questions (Table 1). Question categories include participant contact information, organization information, demographics, professional practice, experience, motivation for participating, self-efficacy, barriers, changes in practice, satisfaction, value of participation, and suggestions.

**Table 1.** Survey questions in previous instruments and minimum dataset.

Surveys and categories of questions	Questions in previous instruments, n	Questions in minimum dataset, n
Presurvey		
Participant contact information	12	13
Organization information	108	8
Demographics^a^	19	16
Professional practice	68	12
Experience	23	3
Motivation for participating	7	2
Presurvey and postsurvey		
Self-efficacy	306	36
Barriers	3	13
Knowledge quiz	104	0
Others, including transition planning and beliefs	38	0
Postsurvey		
Changes in practice	14	5
Satisfaction	25	5
Value of the program	28	7
Suggestions	6	2
Total	761	122

a While there are 53 demographic questions in the minimum dataset survey, a maximum of 16 will be seen by a participant, due to branching logic on the questions. For example, if a participant says they work as a physician, they will only see the physician follow-up questions for specialty, credentials, and their hierarchy (eg, attending, fellow, intern).

The MDS self-efficacy categories, consistent with EAC values, and example statements include the following:

1. Access to equitable best practices
  - Understand best practice screening tools to assess characteristics of autism specific to your professional role
  - Recognize common co-occurring medical conditions in autism
  - Evaluate risks and benefits of interventions related to autism
2. Person-family-centered care
  - Engage in shared decision-making about next steps for the future
  - Appreciate the influence autism spectrum disorder may have on loved ones of an autistic person
3. Embrace curiosity
  - Discuss controversial topics about autism in a respectful way
  - Respect an alternative viewpoint from your own related to autism
4. All teach, all learn
  - Identify characteristics of autism in children
  - Refute or disprove myths associated with characteristics of autism spectrum disorder
  - Serve as a local “expert” about autism in your community
5. See the possibilities in all abilities
  - Perceive a person on the autism spectrum as capable of a meaningful life
  - Proactively share information about predictable life events to support positive transitions

### Implementation Findings

Pilot data included a 100% response rate to presurvey data (n=38) and confirmed that the target audience was recruited and provided a baseline for participant demographics, prior experience, practice patterns, and self-confidence. Changes to the language used for demographics and practice patterns were guided by pilot data results. Despite requiring answers to all questions, a few participants did not complete full surveys, resulting in inconsistent denominators on survey questions. Our organization’s collective experience showed that presurveys included with registration or as part of a training agreement yield higher response rates; most programs included in this study requested presurvey responses in this manner. Additionally, experience indicated that postsurvey invitations that were required for CE or continuing medical education credit were incentivized with a small (US $25‐50 gift card) token of appreciation, or included as a requirement of a training agreement also yielded higher response rates. Where available within programs, we used these methods to encourage responses.

The 8 programs that began and ended within 2023‐2024 included presurveys sent to 710 registrants. Of these, 399 registrants started the presurvey, and 374 completed it; 764 attendees were sent the postsurvey, 250 started the postsurvey, and 239 completed the postsurvey. New presurveys were not sent to returning participants or to anyone without an email address at first attendance. The team concluded their survey responses would not be true presurveys. Presurvey data include both attendees and nonattendees, as attendance data were identified and kept separately, and this study used deidentified data. Postsurveys were sent to all participants, regardless of presurvey status. Of the 25 presurveys with missing data, 7 occurred due to abandonment, most often following demographic questions and before self-efficacy evaluation. Of the 11 postsurveys with missing data, only one was abandoned. All other missing data were due to skipped questions. Response rates were calculated by program. When the presurvey was included as part of registration or a training agreement, response rates ranged from 54% (7/13) to 100% (22/22 and 20/20, representing 2 surveys). When not included in another registration step, or where registrants were explicitly told they could complete the survey later, presurvey response rates ranged from 21% (40/193) to 36% (36/99). Cohort-based programs—where participant groups were often limited, registration closed before the program began, postsurveys were incentivized, and additional trainings were included—had a higher postsurvey response rate (76%, 19/25 to 77%, 46/60 and 17/22) than noncohort or mixed groups (15%, 46/315 to 51%, 37/73).

Hub teams and the EAC leadership team were provided program-level reports at the beginning of each program, which included all presurvey data for the program, and at the end of the program, which included participant pre- and postsurvey data. MDS questions for all programs were combined and loaded into an interactive PowerBI dashboard, which allowed the leadership team to make comparisons of program participant information and interact with results in aggregate. Analyses across combined programs indicated statistical significance for all self-efficacy categories. Statistical tests completed for each individual program are presented in Table 2.

**Table 2.** Postsurvey self-efficacy for ECHO Autism in 2023‐2024 with Wilcoxon signed-rank tests comparing before and after program participation responses, grouped by program and self-efficacy domain^a^.

Self-efficacy domain and program	Response rate, n (%)	Test statistic (W)	*P* value	Effect size (*r*; 95% CI)	Effect magnitude	Participants who reported no change, n (%)	Percentage change
Domain: Access to equitable best practices							
All ECHO Autism programs	239/764 (31)	0	<.001	0.64 (0.58‐0.69)	Large	11/239 (5)	27.2
Advocates	22/62 (35)	0	<.001	0.66 (0.44‐0.81)	Large	2/22 (9)	23.9
Advanced Diagnosis (includes cohort)	37/73 (51)	0	<.001	0.67 (0.52‐0.77)	Large	0/37 (0)	33.1
Behavior Solutions in Hospitals cohort	46/60 (77)	0	<.001	0.61 (0.46‐0.74)	Large	0/46 (0)	25.6
Center Engagement	25/57 (44)	0	<.001	0.38 (0.09‐0.61)	Moderate	3/25 (12)	9.1
Early Intervention	13/32 (41)	0	<.001	0.56 (0.26‐0.78)	Large	0/13 (0)	17.0
Early Diagnostic cohort	17/22 (77)	0	<.001	0.82 (0.71‐0.86)	Large	1/17 (6)	41.2
Mental Health cohort	19/25 (76)	0	<.001	0.82 (0.72‐0.86)	Large	1/19 (5)	38.7
Missouri Alliance for Dual Diagnosis (MOADD)	19/118 (16)	0	<.001	0.69 (0.47‐0.83)	Large	1/19 (5)	25.6
Primary Care	c46/315 (15)	0	<.001	0.66 (0.52‐0.77)	Large	3/46 (7)	29.5
Domain: All teach, all learn							
All ECHO Autism programs	239/764 (31)	0	<.001	0.59 (0.53‐0.66)	Large	27/239 (11)	24.6
Advocates	22/62 (35)	0	<.001	0.52 (0.26‐0.74)	Large	2/22 (9)	20.4
Advanced Diagnosis (includes cohort)	37/73 (51)	0	<.001	0.66 (0.51‐0.77)	Large	1/37 (3)	33.7
Behavior Solutions in Hospitals cohort	46/60 (77)	0	<.001	0.65 (0.51‐0.77)	Large	6/46 (13)	22.1
Center Engagement	25/57 (44)	0	<.001	0.41 (0.08‐0.68)	Moderate	9/25 (36)	7.2
Early Intervention	13/32 (41)	0	.003	0.36 (0.03‐0.69)	Moderate	2/13 (15)	11.2
Early Diagnostic cohort	17/22 (77)	0	<.001	0.73 (0.54‐0.85)	Large	1/17 (6)	34.8
Mental Health cohort	19/25 (76)	0	<.001	0.76 (0.55‐0.86)	Large	0/19 (0)	37.1
MOADD	19/118 (16)	0	<.001	0.54 (0.26‐0.75)	Large	2/19 (11)	22.4
Primary Care	46/315 (15)	0	<.001	0.65 (0.5‐0.76)	Large	4/46 (9)	27.4
Domain: Embrace curiosity							
All ECHO Autism programs	239/764 (31)	0	<.001	0.6 (0.53‐0.66)	Large	56/239 (23)	21.8
Advocates	22/62 (35)	0	<.001	0.52 (0.26‐0.73)	Large	3/22 (14)	20.1
Advanced Diagnosis (includes cohort)	37/73 (51)	0	<.001	0.66 (0.5‐0.78)	Large	5/37 (14)	26.4
Behavior Solutions in Hospitals cohort	46/60 (77)	0	<.001	0.52 (0.32‐0.67)	Large	12/46 (26)	19.9
Center Engagement	25/57 (44)	0	.003	0.54 (0.2‐0.79)	Large	14/25 (56)	7.0
Early Intervention	13/32 (41)	0	.003	0.61 (0.28‐0.81)	Large	2/13 (15)	18.0
Early Diagnostic cohort	17/22 (77)	0	.001	0.8 (0.65‐0.86)	Large	3/17 (18)	30.1
Mental Health cohort	19/25 (76)	0	<.001	0.77 (0.61‐0.87)	Large	2/19 (11)	29.0
MOADD	19/118 (16)	0	.001	0.68 (0.43‐0.83)	Large	6/19 (32)	18.9
Primary Care	46/315 (15)	0	<.001	0.58 (0.41‐0.72)	Large	9/46 (20)	25.6
Domain: Person-family-centered care							
All ECHO Autism programs	239/764 (31)	0	<.001	0.59 (0.52‐0.65)	Large	31/239 (13)	23.8
Advocates	22/62 (35)	0	<.001	0.55 (0.27‐0.76)	Large	4/22 (18)	18.6
Advanced Diagnosis (includes cohort)	37/73 (51)	0	<.001	0.62 (0.45‐0.75)	Large	2/37 (5)	31.5
Behavior Solutions in Hospitals cohort	46/60 (77)	0	<.001	0.6 (0.45‐0.73)	Large	6/46 (13)	24.4
Center Engagement	25/57 (44)	0	<.001	0.55 (0.26‐0.78)	Large	8/25 (32)	9.6
Early Intervention	13/32 (41)	0	.003	0.48 (0.11‐0.76)	Moderate	2/13 (15)	13.1
Early Diagnostic cohort	17/22 (77)	0	<.001	0.71 (0.52‐0.84)	Large	1/17 (6)	31.8
Mental Health cohort	19/25 (76)	0	<.001	0.77 (0.62‐0.85)	Large	1/19 (5)	32.4
MOADD	19/118 (16)	0	<.001	0.64 (0.38‐0.83)	Large	3/19 (16)	19.7
Primary Care	46/315 (15)	0	<.001	0.6 (0.43‐0.75)	Large	4/46 (9)	26.0
Domain: See the possibilities in all abilities							
All ECHO Autism programs	239/764 (31)	11	<.001	0.61 (0.54‐0.68)	Large	63/239 (26)	20.3
Advocates	22/62 (35)	0	<.001	0.6 (0.28‐0.82)	Large	9/22 (41)	13.6
Advanced Diagnosis (includes cohort)	37/73 (51)	0	<.001	0.64 (0.47‐0.76)	Large	7/37 (19)	25.2
Behavior Solutions in Hospitals cohort	46/60 (77)	0	<.001	0.59 (0.4‐0.73)	Large	9/46 (20)	23.0
Center engagement	25/57 (44)	2	.008	0.39 (0.03‐0.72)	Moderate	15/25 (60)	5.3
Early Intervention	13/32 (41)	0	.005	0.7 (0.44‐0.85)	Large	3/13 (23)	13.5
Early Diagnostic cohort	17/22 (77)	0	.002	0.72 (0.5‐0.84)	Large	4/17 (24)	26.0
Mental Health cohort	19/25 (76)	0	<.001	0.69 (0.5‐0.82)	Large	0/19 (0)	26.3
MOADD	19/118 (16)	0	.003	0.61 (0.31‐0.81)	Large	8/19 (42)	13.6
Primary Care	46/315 (15)	0	<.001	0.66 (0.51‐0.77)	Large	8/46 (17)	25.8

a The Wilcoxon statistic and *r* were calculated on the nonzero difference, calculated as (n – participants who reported no change).

Since all Wilcoxon signed-rank tests were significant and effect sizes were all moderate or large, when comparing programs, it is valuable to look at the number of participants who reported no change in self-efficacy and the response rates. A comparison highlights the differences between noncohort programs and cohort groups (ie, Advanced Diagnosis, Behavior Solutions in Hospitals, Early Diagnosis, and Mental Health), where more participants completed surveys and most indicated a change in self-efficacy.

**Figure 2.**
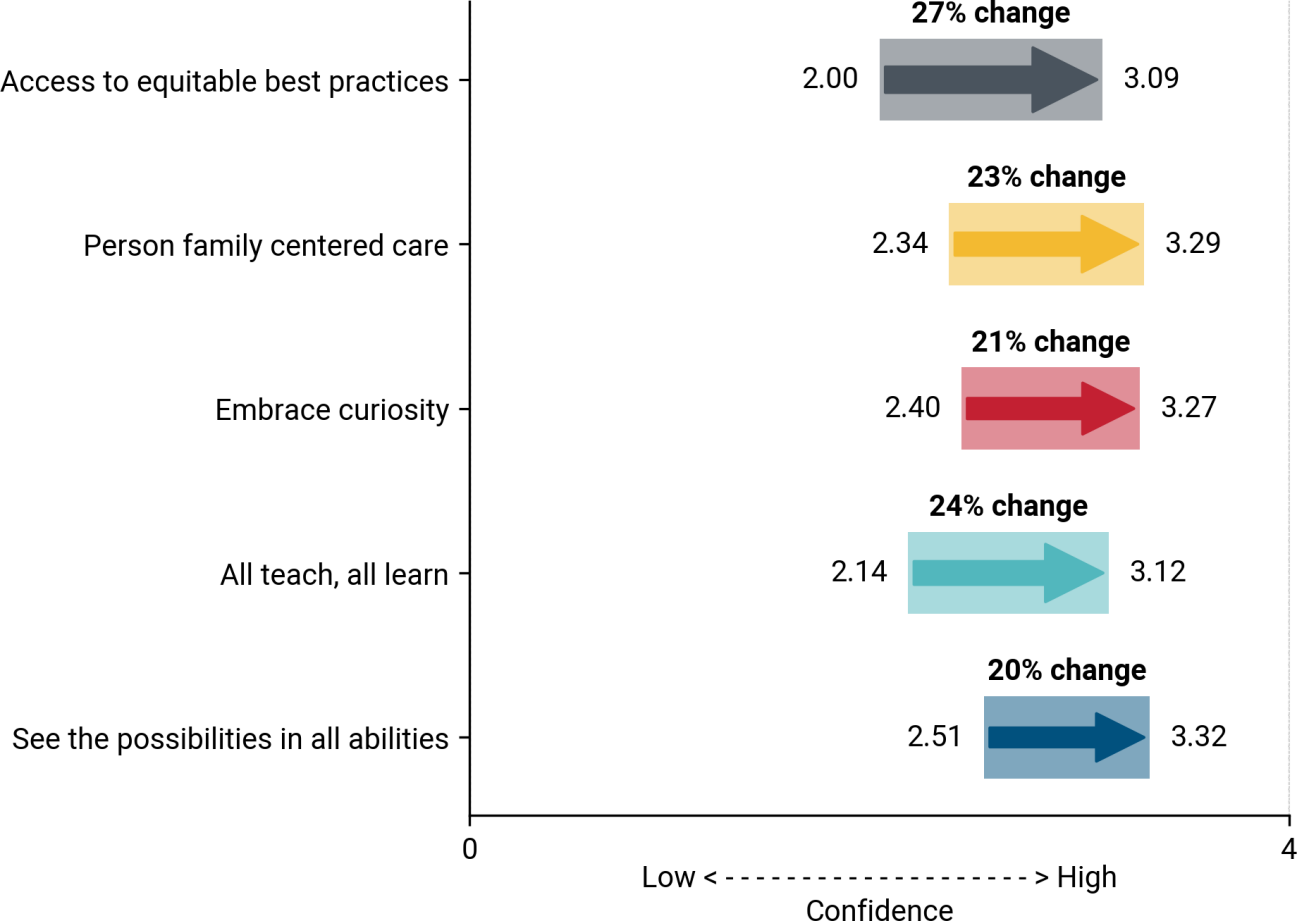
Data display of change in self-efficacy across 8 ECHO (Extension for Community Healthcare Outcomes) Autism programs in 2023‐2024 (n=239), where the left and right edges show average self-efficacy before and after participation and bars show the change.

Self-efficacy visuals are needed to allow viewers to discern the average participant pre/post confidence as well as the degree of self-efficacy change and are displayed as bar charts with a focus on the percent change (Figure 2). Viewer feedback indicated appreciation for the rapid discernment of the program’s effectiveness by seeing percent change and a simplified confidence axis.

## Discussion

### Principal Findings

The ECHO Autism MDS developed in this study was designed to standardize assessment of programs, streamline data analysis, and support organization-wide evaluation. The results of the study indicate that the MDS provides rapid, valuable, and actionable information about the whole organization. Refined data displays result in actionable information for expert hub team members and leadership to assess programs. The MDS draws a line of sight between organization values and program results and meets the goals of the study.

### Comparison to Prior Work

Prior to this study, data were collected at a program level and adjusted at each iteration, limiting cross-program aggregates and comparisons. Programs collected presurveys in varied ways, which reduced response rates in some cases where too little data existed to analyze. Programs did not consistently include retro-pre assessment at post. When response rates were low and no retro-pre was included, a paired analysis became unreliable due to the removal of responses at both sides of the pairing. As a result of the development of standard metrics, consistent data collection methods, using a retro-pre so that all post data could be analyzed, and the implementation of consistent visuals and analysis, ECHO Autism is better able to produce rapid reports and conduct cross-program and longitudinal evaluation.

### Lessons Learned

One of the insights from our pilot was the importance of ensuring each question addresses only one concept. During the pilot, we discovered we had inadvertently combined some questions that should have been separated. Because of our pilot, we were able to split unclear metrics into multiple questions before implementing across all programs and sharing with our partners.

The study team intentionally left a few challenging questions open-ended until more data could inform next steps. One challenging question was ethnicity. While it is a commonly asked question, ethnicity is often confused with race. Our review of other surveys found a range of existing answer choices for this question, including ancestral, geographic, or contemporary group choices. As an example, the US Census survey presents ethnicity as a binary Hispanic/Latino choice [30]. This was not sufficient for our global partners. We used language that would allow people to define their own ethnicity. A preliminary review of data received on this question confirms the confusion. Most responses were a repeat of race answers. Some responses were related to Hispanic identity, such as the US Census. Work to create an appropriate and inclusive ethnicity question is ongoing.

Another challenge that we found in development for a global audience is that of job titles and credentials, and to a lesser degree, highest education. In the English version of the MDS, a respondent chooses a field of work from a set of categories, and then they choose a role and credentials from a list related to their field of work. In the Spanish version, feedback indicated this approach was confusing. The Spanish version of the MDS removes the field of work hierarchy and provides only an open field for credentials. Credentialing is less common in Latin America; thus, this data field is not required. Highest education was translated with an attempt to align years of education to the US standard levels of education rather than verbatim.

The study team also reviewed participant self-identified barriers to working with autistic individuals and their families. In the pilot, the question was asked with a list of “check all that apply.” After pilot data analysis, we implemented a more nuanced approach with a Likert scale to better support evaluation of a change in barriers. The updated MDS uses a 4-point Likert scale from not a barrier to severe barrier.

### Limitations

The study team considered the development of the MDS in relation to Donald Kirkpatrick’s 4-level evaluation model, a framework for assessing training programs. Kirkpatrick’s four levels include (1) how participants feel about the training (reaction), (2) what knowledge or skills a participant learned (learning), (3) how participants applied the learning to their job (behavior), and (4) the impact of the program (results) [31]. When framed in the context of Kirkpatrick’s 4-level evaluation model, EAC program measures included aspects of all 4 levels, but inconsistently. All programs measured reaction (level 1), and some programs measured knowledge (level 2). In developing the MDS, the study team sought to move toward a measure of changes in practice, which requires evaluating behavior (level 3) [31]. For level 3 evaluation, we recognize participant self-assessment is not as robust a measure as consistent and unbiased behavioral data; however, we determined the value of self-assessment as an indicator of progress that we would not otherwise have consistently across programs.

Similarly, we recognize that the use of retro-pre to allow respondents to define how much they felt their self-efficacy had changed has limitations. This design has known biases, including recall distortion, susceptibility to social desirability, and implicit theories of change that may result in inflated results [23]. However, as long as ECHO Autism programs collect pairable self-efficacy data in the presurvey (true-pre format) as well as retro-pre, the same methods of analysis could be used for the true-pre data with the post data in future analyses. We believe this allows for more flexibility and opportunity to analyze for possible biases.

We also recognize that nonresponse bias may exist in the survey data. Previous studies have shown that positive responses could be inflated due to a higher likelihood of pleased participant responders [32,33]. While we did not formally review attendance and other surveys alongside end-survey response rates in this study due to the deidentification of the data, an analysis of response rate, demographics, and frequency of attendance would presumably illuminate whether nonresponse is higher among subgroups of participants. This analysis would provide additional evidence to indicate whether participants self-select to complete surveys based on how pleased they are with the program.

In the “Introduction” section, we discussed challenges we anticipated to standardizing learning and behavioral measures across substantially different programs, including the changing landscape of best practices, program-specific curriculum differences, and a lack of standard observable behavior change data collection methods. Thus, it was determined that level 2 evaluation of direct learning was not a primary focus of the EAC standardized measures, but individual programs could develop and modify knowledge quizzes (level 2 evaluations) yearly. With oversight and review by the hub team, during this study, we modified quizzes for 2 programs to ensure that the questions were all multiple choice with only one correct answer and three incorrect, including only best practices, and the information was still contained in the teaching. Participants completed the quiz within both the pre- and postsurveys, and the results were presented only within the series-level evaluation PDF reports. By EAC leadership request, we removed the knowledge quizzes from these programs for later series because of the additional length added to the surveys and demands on the hub team’s time to review and update the knowledge quizzes for each series.

Beyond the pilot study, and testing by ECHO Autism team members, no usability and technical function testing occurred due to time constraints. As evidenced by the postsurvey self-efficacy results, most participants identified that their confidence increased. While self-reported data are useful, we recognize the limitations and acknowledge there are still opportunities for advancing toward the fourth level in the Kirkpatrick model.

Despite limitations, a wealth of information exists within self-reported data. The hub team provided aggregate results for each statement within the values-based categories for more detailed exploration, and additional self-efficacy statements are typically included to measure program-specific outcomes. As discussed in the “Introduction” section, for many of our participants, no other standardized directly measured behavior change information exists that crosses professional domains.

Future work to validate the psychometrics including participants with global partners would address shortcomings and strengthen the feasibility and usefulness of these standardized measures. Additional work to create unbiased behavioral change assessment through additional measures may also be conducted to further improve the program assessment.

### Conclusions

Previous anecdotal evidence and comments from participants of EAC programs indicated the ECHO model is an effective method for delivering high-quality information to focused entities to create positive change in dispersed communities where access to specialized care is limited. This study sought to create standardized self-reported measures to provide more systematic evaluation of ECHO Autism programs. The ECHO Autism Communities MDS allows us to (1) measure across all sites and programs, (2) set consistent scales and anchors, (3) use inclusive language, and (4) streamline administrative support for administration and analysis. Since we began offering the MDS to ECHO Autism partners in 2023, it has been downloaded by 11 partners and also used by at least 9 partners in 7 countries. We have planned for periodic review and improvement cycles for the MDS and are committed to seeking higher levels of analysis on the effectiveness of ECHO Autism Communities programs. While we seek to develop additional measures to assess programmatic success and participant growth, these self-reported data lay the foundation for cross-program evaluation. Our next steps include using the results for decision-making, reflecting on results, and refining the measures to meet our goals for evaluating our programs and organization. The continued collection of the MDS and expansion of other programs using the measures will support longitudinal evaluation and provide the basis for data-informed program changes and support for systematic change.

## Supplementary material

10.2196/86274 Multimedia Appendix 1 ECHO Autism Minimum dataset postsurvey self-efficacy data collected from 2023 to 2024 across 8 programs.

## Abbreviations

AHRQ: Agency for Healthcare Research and Quality
CE: continuing education
EAC: ECHO Autism Communities
ECHO: Extension for Community Healthcare Outcomes
IRB: Institutional Review Board
MDS: minimum dataset
